# Synthetic population generation with public health characteristics for spatial agent-based models

**DOI:** 10.1371/journal.pcbi.1012439

**Published:** 2025-03-17

**Authors:** Emma Von Hoene, Amira Roess, Hamdi Kavak, Taylor Anderson

**Affiliations:** 1 Department of Geography and Geoinformation Science, George Mason University, Fairfax, Virginia, United States of America; 2 Department of Global and Community Health, George Mason University, Fairfax, Virginia, United States of America; 3 Department of Computational and Data Sciences, George Mason University, Fairfax, Virginia, United States of America; University of Melbourne, AUSTRALIA

## Abstract

Agent-based models (ABMs) simulate the behaviors, interactions, and disease transmission between individual “agents” within their environment, enabling the investigation of the underlying processes driving disease dynamics and how these processes may be influenced by policy interventions. Despite the critical role that characteristics such as health attitudes and vaccination status play in disease outcomes, the initialization of agent populations with these variables is often oversimplified, overlooking statistical relationships between attitudes and other characteristics or lacking spatial heterogeneity. Leveraging population synthesis methods to create populations with realistic health attitudes and protective behaviors for spatial ABMs has yet to be fully explored. Therefore, this study introduces a novel application for generating synthetic populations with protective behaviors and associated attitudes using public health surveys instead of traditional individual-level survey datasets from the census. We test our approach using two different public health surveys to create two synthetic populations representing individuals aged 18 and over in Virginia, U.S., and their COVID-19 vaccine attitudes and uptake as of December 2021. Results show that integrating public health surveys into synthetic population generation processes preserves the statistical relationships between vaccine uptake and attitudes in different demographic groups while capturing spatial heterogeneity at fine scales. This approach can support disease simulations that aim to explore how real populations might respond to interventions and how these responses may lead to demographic or geographic health disparities. Our study also demonstrates the potential for initializing agents with variables relevant to public health domains that extend beyond infectious diseases, ultimately advancing data-driven ABMs for geographically targeted decision-making.

## Author summary

In this study, we introduce a new method for generating synthetic populations of individuals or “agents” with characteristics that include health protective behaviors and attitudes, which are crucial for modeling disease spread. Traditional methods for parameterizing agents often overlook the complex relationships between demographic factors and health behaviors like vaccination. Additionally, detailed spatial data capturing these behaviors are limited, meaning agent behaviors are represented more uniformly across geographic space. By fitting public health surveys with spatially aggregated census data, we created agent populations that reasonably reflect real-world populations for disease spread simulations. We focused on Virginia, U.S. and generated a population with COVID-19 vaccine uptake and attitudes as of December 2021. Our results show that this approach captures the statistical relationships between demographic variables and vaccine uptake, along with the spatial variation in these behaviors. The approach is flexible so that it can be applied to various public health studies beyond just infectious diseases. Our work highlights the potential of public health surveys for enhancing synthetic population generation, offering a valuable approach for initializing models with more realistic populations to explore public health challenges.

## 1. Introduction

Agent-based models (ABMs) are commonly used to simulate the spread of infectious diseases between individuals, including COVID-19 virus [1–4], influenza [5,6], and the chickenpox virus [7,8]. Unlike traditional compartmental models that use differential equations to predict the proportion of the population that is Susceptible, Infectious, and Recovered at each time step (e.g., SIR, or other variants of such models), ABMs use a bottom-up approach that simulates the behaviors, interactions, and subsequent transmission of disease between individual “agents” within their environment [9,10]. This approach allows for the investigation of the underlying processes driving disease dynamics and how these processes may be influenced by policy interventions [11].

Given the important role of demographic characteristics such as age and income [12,13], household structures [14–16], activity patterns, and co-location [17] in disease dynamics, most ABMs of disease spread attempt to incorporate these attributes when initializing agents. For example, children often participate in activities like attending school or recreational events, where they interact with many other individuals and are more likely to contract pathogens that can then be transmitted to parents or grandparents living in the same household [18]. While some studies use random functions or fixed values to assign agent attributes, population synthesis approaches can utilize spatially aggregated census data and individual-level survey data [19] from sources like household travel surveys [20] or census microdata [21]. These methods enable the creation of a complete agent population with relevant attributes, including household structures, to accurately capture transmission pathways within the model.

Disease dynamics are also shaped by the uptake of protective behaviors within the population, such as wearing masks, getting vaccinated, and staying home when sick, which can reduce the likelihood of negative health outcomes [22]. In addition to social norms and physical or financial barriers, an individual’s attitudes, beliefs, and perceptions significantly affect their decision to engage in protective behaviors. Although traditionally overlooked in ABMs of disease spread [23–25], the COVID-19 pandemic spurred on a widespread effort to better represent health behaviors and their dynamics into epidemiological models [26–29]. This paper argues that a synthetic population generation approach capable of initializing agent populations with a realistic set of attitudes and protective behaviors can support such ABMs that aim to simulate behavior dynamics influenced by these attributes.

A typical approach in current ABMs involves assigning protective behaviors or related attitudes to agents based on some probability, using either hypothetical scenarios or aggregated data measuring the real characteristics of the population. For example, Rafferty et al. [7] use an ABM to simulate the impact of dose timing, coverage, and waning of immunity on chickenpox disease outcomes in Alberta, Canada. They initialize the population with vaccination attitudes based on aggregate data (65% acceptance, 30% hesitant, 5% reject). However, this approach ignores the statistical relationships between vaccine attitudes and other individual demographic, cultural, or political characteristics that synthetic population generation approaches aim to preserve.

In another example, Pandey et al. [30] use an ABM to examine the effect of bivalent boosters on COVID-19 outcomes, assuming a coverage of 59%, 51%, 38%, 54%, and 75% for age groups 5-11, 12-17, 18-49, 50-64 and 65+, respectively, informed by historical influenza data. While their model more accurately captures the relationship between booster coverage and age, whereby age 65+ are more likely to accept a booster, the study assumes spatially uniform uptake across New York City. This assumption of uniformity is common, especially since health data is often not available at finer granularities than county or state levels, meaning that spatial heterogeneities can only be captured at these coarser scales. While numerous ABMs have been developed to simulate the adoption of protective behaviors or the spread of beliefs, attitudes, perceptions towards vaccines over space and time, the use of synthetic population approaches to initialize an agent population with these characteristics has yet to be explored.

Therefore, the purpose of this study is to investigate how synthetic population generation approaches can be expanded to create agent populations with attitudes and initial adoption of protective behaviors, along with their spatial distributions. Specifically, we aim to replace datasets commonly used in synthetic population generation that provide individual-level data from samples with coarser geographic resolution, such as the U.S. Census Bureau’s Public Use Microdata Sample (PUMS) [21], with public health surveys. Using COVID-19 as a case study, we explore the potential for this approach by generating a synthetic population representing Virginia, U.S. and their vaccine attitudes and uptake as of December 2021. We obtain real vaccine uptake for Virginia at the census tract level for the same point in time to validate our results, comparing the populations generated by two different national public health surveys.

## 2. Background

With the growing use of ABMs across disciplines such as economics, geography and biology [10], a wealth of synthetic population generation methods have been developed to create agent populations. These populations serve as simplified microscopic representations of the targeted population, reflecting individuals and their socio-demographic characteristics relevant to the study [31]. The emergence of synthetic population generation approaches is largely due to several factors, including privacy restrictions that prevent access to detailed individual-level data at fine spatial scales, the ability of ABMs to simulate social dynamics and behaviors which are connected with individual attributes, and advancements that have made ABMs more data-informed and effective as predictive tools for decision support [32,33].

Synthetic population generation methods vary in complexity and are broadly categorized into two approaches: Combinatorial Optimization (CO) and Synthetic Reconstruction (SR) [34]. CO focuses on replicating real entities by reweighting an existing dataset to match individual profiles. In contrast, SR, which is more commonly used and well-established, generates populations through random sampling from known distributions of demographic characteristics or estimated joint distributions using deterministic re-weighting algorithms like Iterative Proportional Fitting (IPF) [31,35]. Recent years also have seen a rise of approaches based on contemporary techniques, including Markov Chain Monte Carlo [36], Bayesian Networks [37], Hidden Markov Models [38], and Generative Adversarial Networks [33]. Additionally, developed tools like SPEW [39] and Gen* [40] further demonstrate the variety of methods available for synthetic population generation. Given the extensive literature on population synthesis, we provide only a brief background to support understanding of our proposed method. For a comprehensive review of population generation approaches for ABMs, see Chapuis et al. [31].

IPF is the most widely used approach for generating synthetic populations due to its long-standing presence and reliability in literature, computational efficiency, and its methodological simplicity [41]. The algorithm adjusts each cell in an n-dimensional matrix, which represents the distribution of attributes, based on known marginal controls. It starts with sample data to initialize the matrix and then iteratively updates the cells to match the specified contingency dimensions [42]. Originally introduced by Deming and Stephan [43] to adjust contingency tables to fit with known marginal distributions, IPF has been extensively refined by researchers to improve its application for population synthesis. For instance, Beckman et al. [44] first established the methods for using IPF with PUMS data, where joint distributions of household attributes were derived by integrating sample frequency tables from PUMS data with marginal distributions from Census Summary Files, and then randomly selecting households based on these estimates to create a synthetic population.

Synthetic population generation approaches, such as those using IPF to initialize an agent population within a spatial ABM, typically combine spatially aggregate and disaggregated individual-level data to statistically match both the joint distributions found in the individual-level data with the marginal totals in the spatially aggregate data [19]. Spatially aggregate data captures marginal totals of populations across a set of categories such as gender, age, and race within different geographic zones (e.g., census tracts, dissemination areas) in a study area. This data allows for analysis of populations and their spatial distributions with relatively fine granularity while preserving privacy by presenting only marginal totals (e.g., total population aged 65+, or total population that is white) rather than joint distributions across multiple attributes (e.g., total population aged 65+ and white). Disaggregated individual-level survey data contains samples of anonymized records of real individuals and their sociodemographic characteristics. Although this data captures the joint distributions among individual attributes, it represents only a small sample from a large geographic area (e.g., a state or the entire country), which protects individual identities and prevents inference of the spatial distribution of the sample population. Examples of such datasets include PUMS in the U.S., and similar datasets available in other countries, such as Public Use Microdata Files (PUMFs) in Canada.

Synthetic population generation, particularly SR approaches, involves both fitting and allocation. During the fitting stage, IPF is used to align individual-level sociodemographic data with spatially aggregated constraints, generating fractional weights for entities such as households or individuals in each geographic zone. Because IPF outputs fractional weights, allocation is required to produce a discrete set of agent counts that replicates individuals [32]. The fractional weights are converted into integer weights through a process known as ‘integerisation’, which can be performed using various approaches such as simple rounding, thresholding, proportional probabilities, or truncate, replicate, sample (TRS) (see Lovelace and Ballas [45] for a review on these methods). ‘Integerisation’ is followed by expansion, where each individual is represented as a record with a geographic zone, and the matching attributes for that individual from the original survey dataset are carried over [45].

IPF has been used to create agent populations in various spatial ABMs, such as those for disaster management and recovery [46], though it is most commonly used in urban and transportation modeling. However, in the context of spatial ABMs for infectious disease spread, there are few dedicated population synthesis methods or studies utilizing well-established techniques such as IPF [17,47]. This is likely because ABMs take significant time to develop and are often designed for specific objectives, such as understanding the impact of policy guidelines and health behaviors on infectious disease dynamics [42–44,48], proposing general or behavioral frameworks for epidemiological models [45,46,49–53], or forecasting disease transmission [54,55]. This gap is particularly significant as these models are valuable for informing policy, yet generating populations with detailed individual characteristics and health behaviors often remains overlooked, despite their critical role in influencing disease transmission. To our knowledge, no specific synthetic population generation method for spatial ABMs of disease spread has yet been developed to capture both individual attitudes and initial adoption of protective behaviors, along with the spatial heterogeneities in these characteristics. Therefore, there is a need for a flexible synthetic population generation approach that realistically initializes agent populations with attitudes, beliefs, perceptions and initial adoption of protective behaviors, as well their spatial distributions. By proposing a targeted population synthesis method that derives these individual attributes from public health surveys, this approach can be adapted for various public health applications, including infectious diseases, smoking, and other health challenges, across different scales and locations.

## 3. Materials and methods

**Fig 1:**
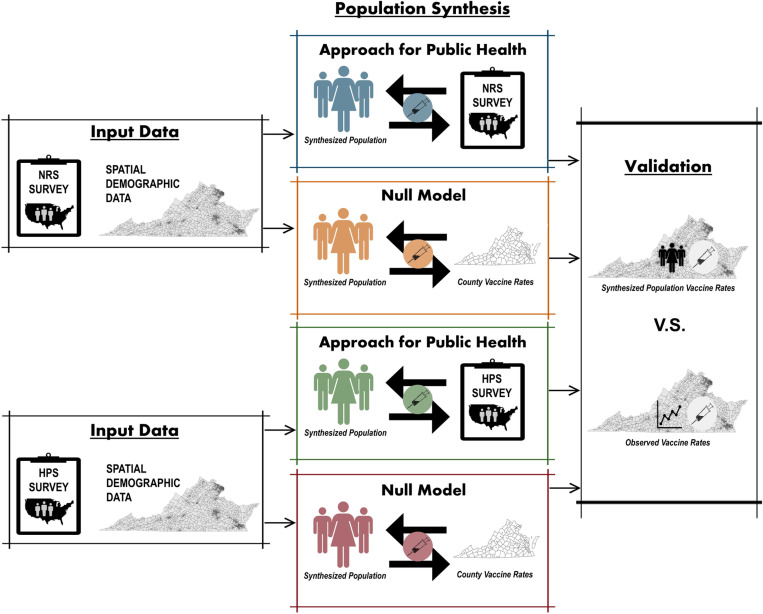
Overview of the approach used in the study. Two survey datasets were used — a Nationally Representative Survey (NRS) conducted by researchers and the Household Pulse Survey (HPS) by the U.S. Census Bureau — along with demographic data for Virginia census tracts from the U.S. Census Bureau. These datasets were integrated with our proposed approach and a null model to synthesize four agent populations representative of Virginia, U.S., which were validated against real-world Virginia census tract vaccination data. The icons within the figure were obtained from open sources, including Openclipart (https://openclipart.org/) and Wikimedia Commons (https://commons.wikimedia.org/wiki/Main_Page). Maps in the figure were created using ArcGIS Pro and openly available boundary files from the U.S. Census Bureau (https://www.census.gov/geographies/mapping-files/time-series/geo/carto-boundary-file.html).

Our approach is presented in Fig 1. First, individual-level survey data and spatially aggregate data are used as input data for the population synthesis of agents with demographic characteristics. Our approach extends traditional synthetic population generation approaches by allowing for vaccination status and attitudes to be carried over at the replication stage. We compare our approach with a null model, which uniformly assigns vaccine uptake likelihood based on county level vaccine uptake data. Our validation involves comparing the vaccination rates of the synthetic population with those of the real population aggregated at the census tract level. This comparison is conducted for populations generated using two different public health surveys and their respective null models. The data and the methods are described in detail in the following sections. The code written in the R scripting language, as well as the data for the synthetic population generation approach and the validation is available at the GitHub repository: *https://github.com/evonhoene/Population-Generation-for-Public-Health-ABMs.*

### 3.1. Inputm data

Given that IPF is a well-established, efficient, and straightforward method for synthetic population generation, we use it to ensure the flexibility of our proposed approach for various study applications. This method requires both spatially aggregated demographic data and individual-level survey data. For the spatially aggregated data, we use census tract data from the U.S. Census Bureau’s American Community Survey (ACS) [56] that captures marginal totals for sociodemographic variables. We focus on gender, race, age, education, and income variables for individuals aged 18 and over, as these factors significantly influence COVID-19 vaccine uptake [57]. While the ACS provides marginal totals for individuals across different categories of gender, race, age, and education, income data is reported as the percentage of households within each census tract that fall into specific income brackets. To assign income to agents, we therefore assume each of our agents as having a household size of 1. While this is a limitation, it is our solution for incorporating income into our synthetic population, an important predictor of vaccine uptake. We use 2021 data specifically for Virginia census tracts and exclude records with missing or zero values for any variable, resulting in a final dataset of N = 2,162. Descriptive statistics for the variables collected from the ACS dataset are presented in Table 1.

**Table 1:** Descriptive statistics summarizing the demographic distribution within Virginia census tracts.

Variable: Descriptor	Mean %	Minimum %	Median %	Maximum %
Gender: Male	48.70285	0	48.73321	100
Gender: Female	51.29715	0	51.26679	100
Race/Ethnicity: White	63.14219	0	66.38809	100
Race/Ethnicity: Black	19.90094	0	12.77431	100
Race/Ethnicity: Hispanic	7.914201	0	5.032508	80.2409
Race/Ethnicity: Other	9.065319	0	5.853839	62.07447
Age: 18-29	20.41858	0	17.94349	98.78631
Age: 30-49	33.61571	0	33.13478	71.48159
Age: 50-64	25.43375	0	25.9862	100
Age: 65 and over	20.53196	0	19.91834	100
Education: Bachelor’s degree or higher	17.29956	0	14.93085	53.30806
Education: No Bachelor’s degree	82.70044	46.69194	85.06915	100
Income: Less than $25,000	14.95912	0	11.69449	100
Income: $25,000 - $49,999	17.3773	0	17.09721	63.68978
Income: $50,000 - $99,999	28.74916	0	28.9908	100
Income: Greater than $100,000	39.18442	0	35.24403	100

Our approach replaces the traditional individual-level samples captured by censuses commonly used in synthetic population generation (e.g., the PUMS in the U.S.) with data from public health surveys. We compare the results of our approach using two surveys, as follows:

1) *Nationally Representative Survey (NRS) Collected by Researchers*: This survey, collected by researchers, is nationally representative and includes data on demographics as well as beliefs, attitudes, and perceptions related to COVID-19 and protective behaviors. The sample was recruited by Climate Nexus Polling (August 15-31, 2021), using several market research panels. Participants were recruited using stratified sampling methods. Compensation for participants depended on the specific market research panel and respondents’ preferences (e.g., cash, gift cards, reward points). Sampling weights accounted for small deviations from the pre-selected census parameters. The dataset includes N = 3,528 respondents. The descriptive statistics for the data are provided in Table 2. De-identified data are made available at the GitHub repository: https://github.com/evonhoene/Population-Generation-for-Public-Health-ABMs. This project to collect the nationally representative survey data was considered exempt by the George Mason University IRB (IRB 1684418-3).
2) *Household Pulse Survey (HPS)*: This publicly available survey, while national, is not stated as representative. The survey was obtained from the U.S. Census Bureau [58], and measures the impact of emergent social and economic issues on households across the country, including COVID-19 vaccinations. The HPS also collects data on core demographic characteristics from respondents aged 18 and older. We use data from HPS Week 41, covering December 29, 2021, to January 10, 2022. Records missing data for one or more variables were removed (e.g., vaccine decision, household income), resulting in a total of N = 63,180 respondents. As described in Table 2, the HPS data shows a bias, with 91.19% of respondents reporting being vaccinated. At the same time, publicly available county-level vaccination data from the CDC [59] indicates that only 50.2% of Virginians were vaccinated by December 30, 2021. To address this bias and reduce the dataset to approximately 3,500 records (aligning with the size of the NRS dataset) by using stratified sampling to create a balanced dataset with 50% vaccinated individuals while preserving demographic representation. The data was split into vaccinated and unvaccinated groups, and strata are created for each combination of demographic variables within each group. Proportional sampling of the survey records was performed within each stratum for both groups, ensuring that the final sample maintained the demographic distribution of the original survey while aligning with CDC-reported vaccination rates in Virginia.

**Table 2:** Comparative distribution of respondent characteristics from the individual-level surveys.

Variable: Descriptor	% Respondents from HPS	% Respondents from NRS
Gender: Male	41.28	44.76
Gender: Female	58.72	55.24
Race/Ethnicity: White	75.06	74.12
Race/Ethnicity: Black	7.01	10.15
Race/Ethnicity: Hispanic	9.16	10.86
Race/Ethnicity: Other	8.78	4.88
Age: 18-29	9.15	16.10
Age: 30-49	36.93	39.12
Age: 50-64	29.15	20.29
Age: 65 and over	24.76	24.49
Education: Bachelor’s degree or higher	41.86	36.96
Education: No Bachelor’s degree	58.14	63.04
Income: Less than $25,000	11.74	52.15
Income: $25,000 - $49,999	19.72	
Income: $50,000 - $99,999	31.03	28.66
Income: Greater than $100,000	37.51	19.19
Vaccination: Yes	91.19	65.33
Vaccination: No	8.81	34.67

Each survey is used to generate a separate set of synthetic population. In the surveys, while some of the individual-level data is measured on a continuous scale (e.g., age), other data are measured categorically, which results in varying levels of detail between the two synthetic populations, depending on the questions asked. For example, when asking about income, the NRS allows respondents to select <$50,000, $50,000-$99,999, and>$100,000. On the other hand, the HPS allows respondents to select <$25,000 and $25,000-$49,999, $50,000-$99,999, and>$100,000, allowing for slightly more detailed agent characteristics related to income. In any case, to be used in the IPF process, the data measured by the individual-level survey must be able to be fall under the categories in the spatially aggregated ACS data. This was possible for attributes including gender, race and ethnicity, age, education, and income. Descriptive statistics for the variables captured by the individual-level surveys and their categories from the two survey datasets are outlined in Table 2.

Our validation approach uses census tract data capturing marginal totals for real vaccine uptake among the total population in Virginia as of December 30, 2021. This data is not publicly available and was acquired by request from the Virginia Department of Public Health. The department has since scaled down its operations, and this data is no longer accessible, even upon request. The vaccine uptake data is available for 1,601 census tracts for which we generate a population. Furthermore, 9 records indicated that vaccine uptake was greater than 100% and were removed. As such our validation only focuses on census tracts where data is available, and vaccine uptake is less than or equal to 100% (N=1,592).

### 3.2. Population synthesis

**Fig 2:**
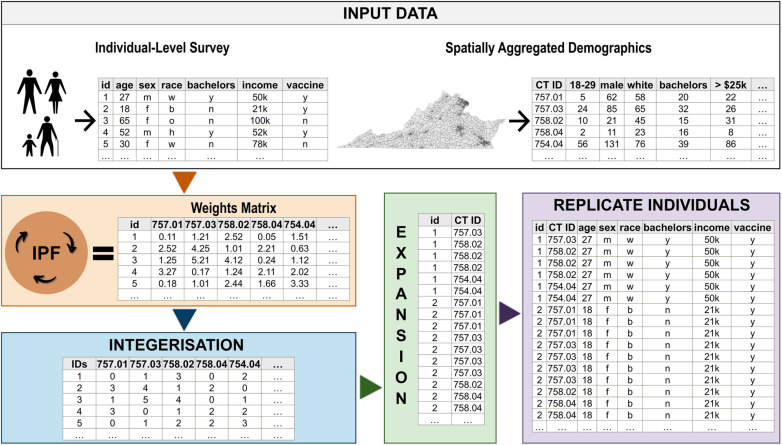
Overview of the synthetic population generation approach for public health applications. Individual-level survey data containing public health characteristics, combined with spatially aggregated demographic data, are processed through an iterative proportional fitting (IPF) method. This generates a weights matrix that is next integerised and expanded to create a final synthetic population dataset. In this dataset, individuals are replicated with health-related attributes and behaviors, such as vaccine uptake, from the survey data. The icons within the figure were obtained from open sources, including Wikimedia Commons (https://commons.wikimedia.org/wiki/Main_Page). Maps in the figure were created using ArcGIS Pro and openly available boundary files from the U.S. Census Bureau (https://www.census.gov/geographies/mapping-files/time-series/geo/carto-boundary-file.html).

***Synthetic population generation approach for public health.*** We use IPF to generate approximately 6 million agents representing the population of Virginia aged 18 and over. This includes generating one population based on the HPS and another using the NRS. Our approach is detailed in Fig 2. IPF computes a weight for every individual in the survey based on how well their characteristics represent the *age, gender, race, income, and education* distributions found in the census tract population. These weights are then processed using the TRS ‘integerisation’ method [45], which involves truncating all weights to integers and using these as the counts of each individual type in the geographic zone, followed by sampling to achieve the correct population size based on the probabilities corresponding to the decimal weights. Simply, this approach converts the weights to integers that describe how many times that individual respondent in the survey should be replicated as an agent in the given census tract. This process is repeated for each census tract in the study area. Following this, expansion is conducted to create the final dataset, where each record corresponds to an individual and their census tract. By replacing traditionally used datasets, such as PUMS, with public health surveys, all or any selected variables captured by the surveys—including demographic characteristics, COVID-19 vaccination status, and attitudes, perceptions, and beliefs related to vaccines—are carried over in the sampling and replication stage.

***Null model.*** We compare the results of our public health synthetic population generation approach with a null model that serves as a baseline. Two distinct populations were generated using the null model, corresponding to the HPS and NRS datasets. With the null model, the IPF method fits the individual level demographic data from the surveys with the census tract data, creating a population of agents with age, gender, race, income, and education characteristics for each census tract in Virginia. However, since vaccine uptake information is only publicly available at county-level, the null model uses this data to impose vaccination uniformly on agents in the same given county. For example, as of December 30, 2021, 84.5% of individuals aged 18+ living in Fairfax County were vaccinated [59]. Therefore, all agents generated in census tracts that fall within Fairfax County in the null model were assigned a vaccination likelihood of 84.5%. This is a common approach in ABM to initialize agents with health variables such as vaccine uptake.

***Validation.*** We compare the spatial and statistical patterns of the simulated vaccine uptake with the observed vaccine uptake percentage at the census tract level and with the individual level survey data for the same time period. Although the population is generated for all census tracts in the study area, validation is only possible for census tracts where real vaccine uptake data is available and where vaccine uptake is less than or equal to 100%.

## 4. Results

We evaluate the observed and simulated percentages of gender, race, age, education, income, and vaccine status variables across Virginia census tracts (N = 1,592) in the populations generated using the HPS and the NRS, using the following quantitative measures: Pearson’s correlation coefficient (r), coefficient of determination (r²), root mean squared error (RMSE), and mean absolute error (MAE).

Pearson’s correlation coefficient measures the strength and direction of the linear relationship between two variables. The coefficient of determination is the square of this coefficient, providing a quantitative measure of how well the variability in one variable is explained by the other. This metric ranges from 0 to 1, where 1 represents a perfect fit and values near 0 indicate little to no association. In this context, r^2^ evaluates how well the patterns in the simulated data, such as vaccine uptake and demographics aggregated by census tract, align with those observed in the real population. Since the IPF approach is designed to fit individual-level data to the marginal totals in census tract data, it is unsurprising that the values of r and r^2^ for gender, race, age, education, and income are very high for both surveys. Because vaccine uptake is typically unavailable at the census tract level and cannot be directly incorporated into the IPF, our approach “carries over” individual vaccine status along with their attitudes, beliefs, and perceptions during the sampling and replication stage (see Fig 2).

**Fig 3:**
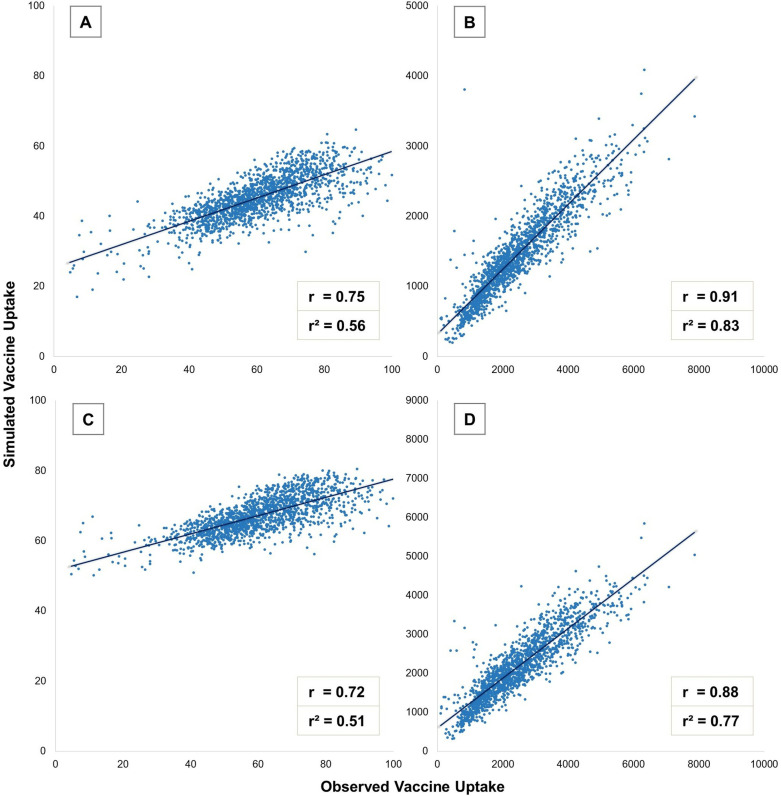
Scatterplots comparing observed and simulated vaccine uptake within Virginia census tracts: A) percentage from the HPS, B) count from the HPS, C) percentage from the NRS, D) count from the NRS.

We find that combining IPF with either of the public health surveys allows us to initialize agents with COVID-19 vaccination status in a way that approximates the real population to a certain extent. The Pearson correlation coefficient and the coefficient of determination for vaccine uptake evaluating the synthetic populations generated from both surveys are moderately high (see Table 3). This is visually depicted in Fig 3, where each scatterplot point represents one of the 1,592 Virginia census tracts, with the x-axis showing the observed percentage of vaccine uptake and the y-axis showing the percentage within the synthesized population. In general, within the simulated population using the HPS with IPF, census tracts with higher real vaccination rates also show higher proportions of vaccinated synthetic individuals, with a moderate positive correlation (r = 0.75, r^2^ = 0.56, Fig 3A). A similar pattern emerges in the simulated population from the NRS (r = 0.72, r^2^ = 0.51, Fig 3B). Additionally, when comparing the count of simulated vaccinated individuals in each census tract to the actual count, we find a stronger positive correlation for the HPS dataset (r = 0.91, r^2^ = 0.83, Fig 3C) and the NRS dataset (r = 0.88, r^2^ = 0.77, Fig 3D). However, this is largely a reflection of how well the IPF simulates the total population in each census tract, as larger populations naturally lead to more vaccinated individuals.

**Table 3:** Evaluation metrics comparing observed and simulated percentages of demographic and vaccine status variables across Virginia census tracts.

Variable: Descriptor	HPS Dataset				NRS Dataset			
	*Pearson’s r*	*r* ^*2*^	*RMSE*	*MAE*	*Pearson’s r*	*r* ^*2*^	*RMSE*	*MAE*
Gender: Male	0.9899	0.9799	0.7784	0.5867	0.9855	0.9712	0.9450	0.7307
Gender: Female	0.9899	0.9799	0.7784	0.5867	0.9855	0.9712	0.9450	0.7307
Race/Ethnicity: White	0.9991	0.9981	1.3229	1.0064	0.9985	0.9969	1.5779	1.2491
Race/Ethnicity: Black	0.9993	0.9987	1.0111	0.6690	0.9990	0.9980	1.1379	0.8145
Race/Ethnicity: Hispanic	0.999	0.9980	0.4696	0.3461	0.9989	0.9978	0.4965	0.3769
Race/Ethnicity: Other	0.9982	0.9965	0.9257	0.5872	0.9985	0.9969	0.5381	0.3612
Age: 18-29	0.9982	0.9963	0.758	0.5076	0.9830	0.9663	2.9901	2.3066
Age: 30-49	0.9948	0.9896	0.9428	0.7392	0.9625	0.9264	3.4143	2.5824
Age: 50-64	0.9945	0.9891	0.7982	0.6127	0.9927	0.9854	0.8424	0.6546
Age: 65 and over	0.9928	0.9857	1.2256	0.9309	0.9963	0.9926	0.7648	0.5728
Education: Bachelor’s degree or higher	0.9969	0.9938	1.7922	1.5619	0.9971	0.9941	2.1185	1.8389
Education: No Bachelor’s degree	0.9969	0.9938	1.7922	1.5619	0.9971	0.9941	2.1185	1.8389
Income: Less than $25,000	0.9992	0.9983	0.5555	0.4177	0.9984	0.9967	1.3171	1.0332
Income: $25,000 - $49,999	0.9984	0.9967	0.6097	0.4674				
Income: $50,000 - $99,999	0.9974	0.9948	0.7428	0.5484	0.9966	0.9933	0.8222	0.6086
Income: Greater than $100,000	0.9994	0.9988	1.0354	0.7246	0.9993	0.9985	1.1931	0.8400
Vaccine Uptake: Received	0.7489	0.5608	18.2780	15.6970	0.7151	0.5113	13.2810	10.6457

RMSE, measured in the same units as the original data, indicates how closely a simulated population matches the actual census tract data, with lower values reflecting a better fit and higher values signaling greater discrepancies. As expected, the RMSE values are low for gender, race, age, education, and income. However, the RMSE for the observed and simulated percentage vaccination rates across census tracts is 18.28 for the HPS dataset and 13.28 for the NRS dataset. These values suggest that, on average, the simulated percentage of vaccinated individuals differs from the actual percentage by 18.28% and 13.28%, indicating a moderate level of inaccuracy.

Similarly, MAE measures the average magnitude of errors between predicted and observed values by averaging the absolute differences, without considering their direction. Unlike RMSE, MAE does not square the errors, making it less sensitive to large deviations and more robust to outliers. MAE values are consistently low for gender, race, age, education, and income variables in both synthetic populations, as IPF effectively fitted these variables to the census tract data. For vaccine uptake percentages, MAE values for the synthesized populations are 15.70 for the HPS dataset and 10.65 for the NRS dataset, which are comparable to the RMSE values. This indicates that the simulated vaccine uptake percentages differ from actual values by 15.70% and 10.65%, respectively, and the similarity between MAE and RMSE values suggests that large deviations do not disproportionately impact the average error. Overall, both RMSE and MAE suggest that the simulated vaccine uptake percentages from our synthetic population generation approach partially reflects the observed values across Virginia census tracts. Furthermore, the RMSE and the MAE are smaller for the NRS dataset. In general, the synthetic populations tend to have a smaller proportion of vaccinated individuals than compared to the real population. This may be because the validation dataset captures vaccination for the total population of Virginia, and we simulate agents aged 18+.

In contrast, the null model shows significantly poorer performance in initializing agents realistically with vaccine decisions, as evidenced by a Pearson correlation coefficient of 0.298 and a coefficient of determination of 0.089. These low values indicate a weak relationship between the simulated and observed vaccine uptake percentages. Additionally, the null model’s RMSE of 30.36 and MAE of 24.01 are considerably higher compared to our proposed approach. These error metrics suggest greater deviations between the simulated and actual vaccine uptake data across census tracts, demonstrating that the null model fails to accurately reflect the real distribution of vaccine uptake. This comparison highlights the limitations of the null model in capturing vaccination behaviors when initializing an agent population and emphasizes the improved performance of our synthetic population generation approach using public health surveys.

Our approach effectively preserves the real-world statistical relationship between sociodemographic variables and vaccine uptake. This is demonstrated by comparing logistic regression coefficients that explain the relationship between these variables and vaccine uptake across the original survey populations, the synthetic populations generated with our approach, and the null model. As shown in Table 4, in the HPS dataset, real individuals who are white, male, or low-income (less than $25,000) have lower vaccination rates (β = -0.2277, -0.0595, -0.5776, respectively), while those who are aged 65+ or hold a bachelor’s degree or higher (β = 1.1670, 1.2429, respectively) are more likely to be vaccinated. The direction and the relative strength of these associations are also reflected in the synthetic population generated using the HPS dataset. In contrast, the null model fails to capture these underlying statistical relationships. For example, in the synthetic population created by the null model, agents aged 65+ are less likely to be vaccinated (β = -0.11). Similarly, while holding a bachelor’s degree or higher is strongly associated with increased vaccine uptake (β = 1.24) in the HPS data, the null model results in a weaker association between education attainment and vaccine uptake (β = 0.24).

**Table 4:** Coefficients from logistic regression models describing the relationship between demographic variables and vaccine uptake from the HPS, along with the synthetic population and null model generated from the HPS. Significant coefficients are indicated with an asterisk (*) at the 90% confidence level (p-value < 0.10).

Variable: Descriptor	HPS Data: R^2^ = 0.08 Coefficient β	Synthetic Population: R^2^ = 0.11 Coefficient β	Null Model: R^2^ = 0.01 Coefficient β
Intercept	1.8615*	- 0.3245*	- 0.2365*
Gender: Male	- 0.0595*	- 0.1872*	0.0025
Race/Ethnicity: White	- 0.2277*	- 0.2758*	- 0.2761*
Age: 65 and over	1.1670*	1.4072*	- 0.1128*
Education: Bachelors or Higher	1.2429*	1.3435*	0.2357*
Income: $25,000 or less	- 0.5776*	- 0.8446*	- 0.3071*

Similar results are presented with the synthetic population generated from the NRS dataset and the corresponding population from the null model (Table 5). In the NRS, individuals who are either aged 65 and older, have a bachelor’s degree or higher, or with high income (greater than $100,000) are more likely to be vaccinated (β = 1.0524, 0.6248, 0.4058, respectively), while individuals who are white are less likely to be vaccinated (β = -0.1511). These associations are reflected in the synthetic population generated using our approach. However, the null model does not capture the strong positive relationship between individuals aged 65+ and vaccine uptake observed in the NRS dataset (β = 1.0524), with the coefficient becoming negative and close to zero (β = -0.0841).

**Table 5:** Coefficients from logistic regression models describing the relationship between demographic variables and vaccine uptake from the NRS, the synthetic population generated from the NRS, and the null model generated from the NRS. Significant coefficients are indicated with an asterisk (*) at the 90% confidence level (p-value < 0.10); Gender was not included due to its insignificance (p-value greater than 0.10) in the NRS dataset.

Variable: Descriptor	NRS Data: R^2^ = 0.05 Coefficient β	Synthetic Population: R^2^ = 0.05 Coefficient β	Null Model: R^2^ = 0.01 Coefficient β
Intercept	0.2341*	0.4020*	- 0.4055*
Race/Ethnicity: White	- 0.1511*	- 0.3036*	- 0.2964*
Age: 65 and over	1.0524*	1.1722*	- 0.0841*
Education: Bachelors or Higher	0.6248*	0.3839*	0.1410*
Income: Greater than $100,000	0.4058*	0.6669*	0.3703*

It is important to note that the logistic regression examples demonstrate how the synthetic populations generated using our public health approach are statistically compared to the real populations from the respective surveys used. Variables for comparison were selected based on their significance in the original surveys, and so gender was excluded from the NRS dataset comparison due to its lack of significance at a 90% confidence level. While the strength and direction of the association between sociodemographic variables and vaccine uptake were preserved in the synthetic populations from both the HPS and NRS datasets, the coefficient of determination (R^2^) for the logistic regression also remained relatively consistent, indicating a similar fit between sociodemographic variables and vaccine uptake. Specifically, the R^2^ was 0.08 for the HPS dataset and 0.11 for the corresponding synthetic population, while it was 0.05 for the NRS dataset and the corresponding synthetic population. In contrast, the null models produced a much lower R^2^ of 0.01.

**Fig 4:**
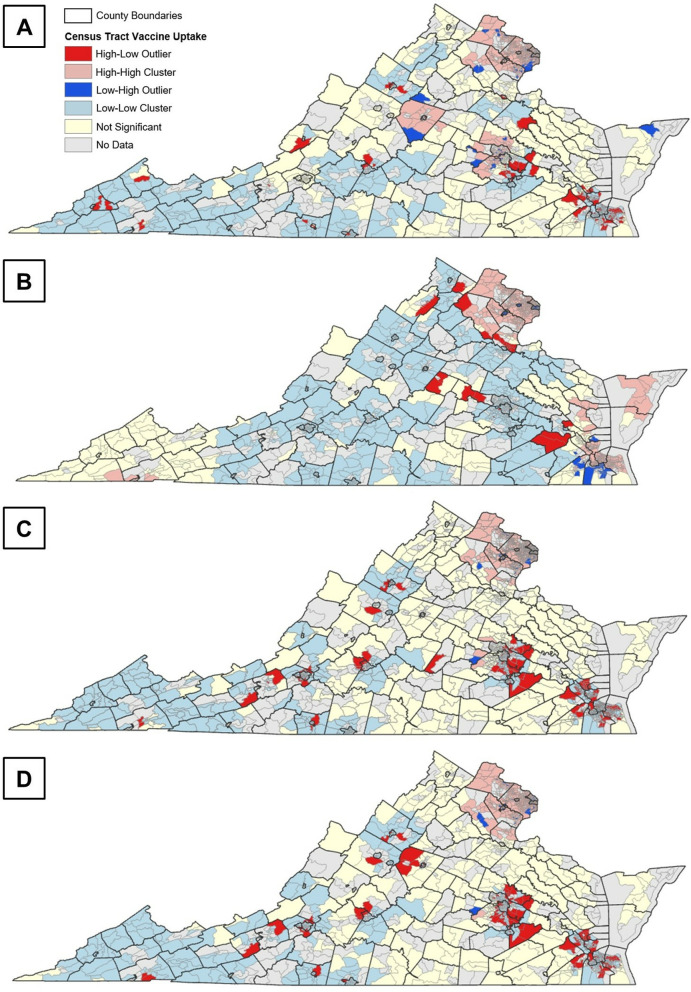
Clusters and outliers of vaccination uptake in Virginia Census Tracts: A) observed, B) null model, and C) synthesized population with HPS data, and D) synthesized population with NRS data. Maps in the figure were created using ArcGIS Pro and openly available boundary files from the U.S. Census Bureau (https://www.census.gov/geographies/mapping-files/time-series/geo/carto-boundary-file.html).

We use spatial autocorrelation metrics, specifically the Anselin Local Moran’s I statistic, to quantify and validate the effectiveness of the proposed population synthesis approach in capturing spatial heterogeneity [60]. A positive I value indicates that a feature is part of a cluster, with neighboring features sharing similar high or low attribute values, while a negative I value suggests that a feature is an outlier, with dissimilar values among its neighbors. In both cases, the feature’s p-value must fall below a specified threshold of 0.05 for the cluster or outlier to be considered statistically significant at the 95% confidence level. The high or low classification is based on whether the percent vaccine uptake of a census tract falls above or below the mean. Fig 4 illustrates the Moran’s I results, showing the spatial distribution of clusters and outliers of vaccination uptake across 1,592 census tracts with data available for both real and synthetic populations. In these maps, a “High-High Cluster” (light pink) indicates that census tracts have high vaccine uptake and are surrounded by counties with similarly high vaccine uptake. In contrast, a “Low-Low Cluster” (light blue) represents census tracts with low vaccine uptake and are surrounded by counties also with low vaccine uptake. Outlier census tracts are identified as “High-Low Outliers” (bright red), where census tracts with high uptake are surrounded by those with low uptake, or “Low-High Outliers” (bright blue), where census tracts with low uptake are surrounded by those with high uptake. Census tracts without a significant relationship to their neighbors are shown in light yellow, while those with no population or available vaccine data are in grey.

The observed vaccine uptake by December 2021 is mapped in Fig 4A. Generally, census tracts in the western part of Virginia show relatively low vaccine uptake. Clusters of tracts with high vaccine uptake are found in Northern Virginia, including Fairfax, Prince William, Loudoun, and Arlington Counties. Other high uptake clusters appear in the central part of the state, such as Albemarle County, which surrounds Charlottesville, and Hanover County, particularly in census tracts west of Richmond. The rest of Virginia exhibits mixed uptake rates, leading to the formation of outliers. These outliers are scattered throughout the state, with many High-Low outliers concentrated in larger areas, such as southeast of the Richmond metropolitan area, around Hampton Roads, and in smaller regions near major cities like Harrisonburg and Forest.

Generally, the population generated using the null model approach captures the spatial heterogeneity of COVID-19 vaccine uptake since the marginal totals of the synthetic population vaccination are imposed to match the real county-level data (Fig 4B). However, given that only county-level data is publicly available, there is less within-county variation. For instance, the null model accurately detects the high vaccine uptake cluster in Northern Virginia but inaccurately suggests similar clusters in the southeastern region and census tracts along the Chesapeake Bay. Additionally, the null model overlooks the low vaccine uptake clusters in southwestern Virginia. It also fails to replicate the general outlier patterns observed in real vaccine uptake (Fig 4A), and specifically misclassifies High-Low outliers in southeastern Virginia as Low-High outliers. This misclassification likely stems from the model’s reliance on uniform county-level vaccine uptake rates, which overlooks finer-scale spatial patterns within counties. Overall, these results suggest that imposing vaccine decisions during the initialization of agent populations does not adequately preserve the spatial distribution of protective behaviors.

The spatial vaccination patterns resulting from the synthetic populations generated using the HPS survey (Fig 4C) and the NRS (Fig 4D) generally align better with observed vaccine rates compared to the null model. They effectively capture the high vaccination cluster in Northern Virginia and the low vaccine cluster in the southwest region of Virginia. However, they fall short in replicating the larger high vaccination clusters in central Virginia near Charlottesville and Richmond. Despite this, our approach excels in preserving both broad regional patterns and location-specific outliers. For example, the High-Low outliers in the Hampton Roads and Richmond metropolitan areas, as well as certain census tracts near Harrisonburg and Forest, are accurately reflected in the synthetic populations. Notably, our method also identifies the sole Low-High outlier census tract west of Richmond, an area nearby many census tracts considered as High-Low outliers. These findings demonstrate the effectiveness of our approach in capturing spatial patterns of protective behaviors, which emerge as clusters or outliers. This aligns with Tobler’s First Law of Geography, which explains how phenomena, such as human behavior, in nearby areas are often more related than those in distant regions [61]. By leveraging these spatial relationships, our approach highlights its potential to support location-specific public health intervention strategies.

**Fig 5:**
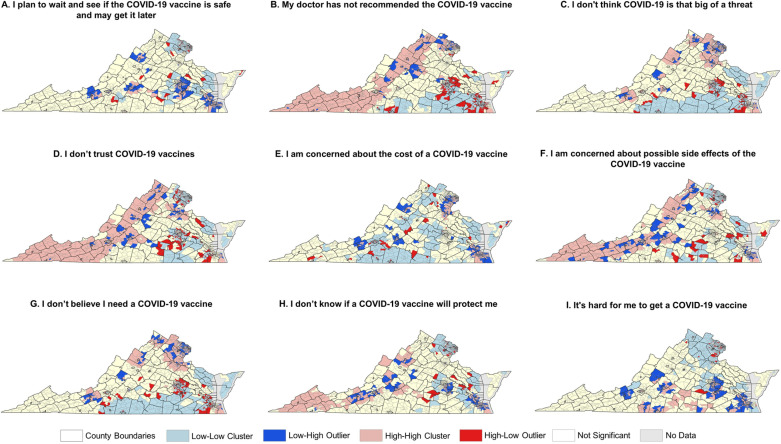
Cluster and outliers of vaccination attitudes, beliefs, and perceptions across Virginia census tracts for the synthetic population generated from the HPS. Maps in the figure were created using ArcGIS Pro and openly available boundary files from the U.S. Census Bureau (https://www.census.gov/geographies/mapping-files/time-series/geo/carto-boundary-file.html).

With our approach, all variables from the public health survey are incorporated into the agent population, enabling us to generate synthetic populations with not only initial uptake of protective behaviors like vaccination but also realistic attitudes, beliefs, and perceptions. This allows for a better understanding of the spatial patterns of these characteristics within a population. Fig 5 illustrates the spatial distribution of vaccination attitudes, beliefs, and perceptions of the synthetic population generated from the HPS survey. For example, in western Virginia, clusters of individuals exhibit vaccine hesitancy due to reasons such as lack of doctor recommendation (Fig 5B), distrust in the vaccine (Fig 5D), or concerns about side effects (Fig 5F). In contrast, Northern Virginia shows a low clustering of individuals planning to wait to see if the vaccine is safe (Fig 5A) or doubting its efficacy (Fig 5H). However, there is a high concentration of individuals who do not perceive COVID-19 as a significant threat (Fig 5C) or do not feel the need for the vaccine (Fig 5G) in Northern Virginia. Concerns about vaccine cost are prevalent in eastern portion of Northern Virginia and extend slightly south, as well as in the southeast around the Hampton Roads region (Fig 5E). Specific census tracts with individuals that believe it is hard for them to get a vaccine are shown in Fig 5I. This approach facilitates the integration of behavioral theories, such as the Health Belief Model (HBM), into ABMs by illustrating how individual attitudes, beliefs, and perceptions affect vaccine uptake and its spatial distribution. This capability ultimately supports the development of ABMs of infectious disease spread that aim to simulate the underlying processes driving the adoption of protective behaviors over space and time, providing a realistic initialization of populations with these characteristics and supporting geographically-targeted interventions.

## 5. Discussion and conclusion

In this study, we investigate the potential to expand synthetic population generation approaches to initialize an agent population with variables relevant for public health, using COVID-19 vaccine uptake as an example. This method enables researchers to quickly initialize a synthetic population where the true statistical relationships between demographic characteristics and public health variables are preserved. Furthermore, the approach captures the spatial heterogeneity of such protective behaviors at finer scales than typically available in spatial data. While protective behaviors such as vaccination, masking, and social distancing can sometimes be found at county or state level, similar data capturing attitudes, beliefs and perceptions that can be simulated using this approach are often not available in spatial data format at all. The synthetic population that was generated using two different surveys, demonstrating the flexibility of the approach to be implemented using a variety of public health surveys. Our results show that such an approach has potential to support disease simulations requiring realistic parameterization of agents with these variables. Beyond simulation, the synthetic populations generated by our approach can be aggregated back to the census tract level, providing novel spatial datasets capturing health-related behaviors, attitudes, and perceptions useful for analyses by decision makers or other scientists.

It is important to note that researchers who have access to fine-grained spatial data capturing health behavior variables (e.g., vaccine uptake at the census tract level) could incorporate that data directly into the IPF approach to more accurately capture the statistical and spatial patterns health behaviors. However, this would be limited to the specific location and time period for which the data is available. For example, our validation dataset captures vaccine uptake at the census tract level for Virginia by December 2021, meaning it could be used in the IPF as another category for which the marginal totals are known. However, this would limit the transferability of the approach to other study areas and points in time. Therefore, we demonstrate how the approach could be implemented using only publicly available longitudinal data such as the HPS, making it straightforward for researchers to generate a synthetic population with these variables anywhere in the country and for multiple points in time.

We also note that researchers have used surveys or other data sources to probabilistically assign vaccine uptake and attitudes based on the agents’ demographics, although this is not the norm and typically only applies different uptake likelihood based on age [30,62]. Such an approach involves using traditional synthetic population generation to create a population of agents with demographic variables and then using separate survey data or other sources to compute and apply the probability that agents within each demographic group will adopt a vaccine. Assuming the same individual-level data is used, this probabilistic approach and our proposed approach will converge towards a similar synthetic population, particularly as the number of combinations of demographic variables considered in the probabilistic approach increase. While both are good approaches, our proposed approach eliminates the need for probabilistic assignment of vaccine uptake and also separately any desired attitudes and perceptions, which requires numerous combinations to achieve a population comparable to our proposed method.

The quality of the synthetic population is limited by the quality of the census tract and individual level survey data. For example, it does not appear that the HPS data is nationally representative and was largely biased towards vaccinated individuals. Therefore, we were able to improve our results slightly using this dataset by adjusting the representation of vaccinated individuals in the sample from 91% (r^2^ = 0.499) to 50% (r^2^ = 0.56), but more research is needed to investigate effects of bias on this approach. Additionally, the ACS census reports household income as the percentage of households within each census tract in specific income brackets, historically limiting IPF approaches from generating individual agents with income characteristics—a key predictor of vaccine uptake. By assuming that individual agents represent single-person households, we effectively leveraged household income data to address this limitation. While we found this approach to be simple and effective, future work may consider other approaches to better estimate individual level income. Furthermore, there are other factors that are likely affecting the individual’s decision to get vaccinated (e.g., policy interventions, social norms) that can’t be directly incorporated into the synthetic population generation approach. Thus, it may be more effective to synthesize a population with vaccine intention, rather than vaccine uptake itself, where such data is available (e.g., using the Understanding Coronavirus in America longitudinal survey).

Overall, this study demonstrates how public health surveys can be used directly in synthetic population generation approaches to initialize a realistic population of agents with vaccine uptake and attitudes. We show that the synthetic populations generated using this approach reflect the real-world statistical relationships between vaccine uptake and different demographic characteristics including race, age, income, education, and gender, while capturing the realistic spatial heterogeneity of such behaviors at the census tract level. While it’s important to strike a balance between complexity and usefulness, it has been shown that realistic agent populations are important for disease simulations. For example, Zhu et al. [17] show that household structures and population demographics play a role in mobility and interaction, and subsequently shape disease transmission dynamics. Even beyond ABM, we note that spatial variation in population characteristics, such as demographic and behavioral attributes, can also be integrated into non-agent-based models of disease transmission to inform decision-making on local exposure risks or interventions [63].

Although simulations that ignore or oversimplify population heterogeneity can provide valuable insights, those that incorporate realistic population structures and their relationships with protective behaviors can enable the exploration of more nuanced questions critical for public health decision-making. For example, such models can be used to forecast health disparities stemming from spatial and demographic heterogeneities in protective behaviors, evaluate the adoption and impact of public health interventions across different groups, assess the effects of targeted strategies, and identify vulnerable populations. Ultimately, our proposed synthetic population generation approach has the potential to enhance the predictive power and realism of disease simulations and provide critical insights into how interventions might play out in real-world settings. This potential will be evaluated in future work that integrates our approach for synthetic population generation and an agent-based simulation of COVID-19 spread.

To our knowledge, this study marks one of the first attempts to extend synthetic population generation approaches using public health surveys to initialize agents with protective behaviors and attitudes relevant to ABMs of infectious disease spread. Future research is needed to see if this approach can be used to initialize other health behaviors and associated perceptions and attitudes (e.g., tobacco use in populations using the CDC’s National Tobacco Survey). We encourage other researchers with access to more fine-grained spatially aggregated data to validate this approach across various public health domains, aiming to improve the parameterization of more realistic agent populations in data-driven ABMs for public health.
